# A Hand-Book of Homœopathic Practice

**Published:** 1882-12

**Authors:** 


A HAND-BOOK OF HOMŒOPATHIC PRACTICE. By George M. Ockford, M. D. Pp. 435. Price, $3.00. Chicago: Duncan Brothers. 1882.

"The main object of this * * * Hand-Book is to present in a concise form practical descriptions of the principal diseases and their treatment."—*Preface.*

Dr. Ockford has given in this little hand-book as good a *condensation of treatment* as any one can give. But we cannot prescribe *successfully* on such condensations. The remarks (p. 297) on local treatment of diphtheria are bad. Such treatment does harm. *Vide,* allopathy.

Any one wishing such a book will find this a good one. And it is a great improvement on the usual printing of Duncan Bros.

